# ‏Impact of Food Habits on Cataract Development Among Adults in Aseer Region, Saudi Arabia: A Retrospective Study

**DOI:** 10.7759/cureus.24878

**Published:** 2022-05-10

**Authors:** Waleed Aldhabaan, Ahmed S AL-Zomia, Lama A Lahiq, Mushary Alqahtani, Shuruq Al-Qahtani, Sulafah Aljohani, Tariq Al-mufarrih, Yazeed S Alshahrani

**Affiliations:** 1 Ophthalmology, College of Medicine, King Khalid University, Abha, SAU; 2 Medicine and Surgery, College of Medicine, King Khalid University, Abha, SAU; 3 Medicine, College of Medicine, King Khalid University, Abha, SAU; 4 Medicine and Surgery, Taibah University, Madinah, SAU; 5 Medicine and Surgery, Batterjee Medical College, Abha, SAU

**Keywords:** cataracts, food, antioxidants, risk factors, association, dietary habits, nutrients intake

## Abstract

Background: Cataracts are the main cause of visual impairment among the aging population, with a high impact on patients’ quality of life. It has been suggested that the antioxidant carotenoids lutein and zeaxanthin may play a role in cataract prevention. Recently, significant evidence has associated abnormal glucose metabolism with an increased likelihood of the development of cataracts.

Aim: The current study aims to assess the relationship between nutrition and cataracts among adults in the Aseer Region, Saudi Arabia.

Methods: A retrospective study was conducted targeting all accessible patients diagnosed with cataracts at Aseer Central Hospital during the period from August 8, 2019, to March 3, 2021, and at Khamis Mushayt General Hospital during the period from June 11, 2018, to March 3, 2021. Data were collected using a pre-structured data collection sheet that covered patients’ socio-demographic data, including age, gender, education, work type, and marital status. Also, the type of cataract diagnosed for the study patients was recorded with associated risk factors of cataracts, including chronic diseases, trauma, eye surgery, and family history. The last section covered patients’ dietary habits and frequency of dietary intake.

Results: A total of 140 patients with cataracts who fulfilled the inclusion criteria were included in the study. Patients' ages ranged from 21 to 65 years, with a mean age of 54.2 ± 12.9 years old. Additionally, 12.1% of the study patients were smokers, 44.3% were diagnosed with hypertension, and 45% were diabetic, which was type 1 diabetes mellitus (T1DM) among 36.5% and type 2 diabetes mellitus (T2DM) among 52.4%. A family history of congenital cataracts was reported among 12.9%, and 12.9% had a history of eye trauma. Moreover, 37.1% of the study patients had vegetables once per week. Eating fruits or having juice was reported as once per week among 40% of the study patients and three times per week among 20%. Finally, 31.4% take nutritional supplements or vitamins.

Conclusion: There is currently an inquiry to endorse or exclude a specific diet or dietary intake that may reduce or even prevent the development and progression of cataracts. It appears beneficial for people to have some antioxidants in their daily food.

## Introduction

Cataracts are the main cause of visual impairment among the aging population, with a high impact on patients’ quality of life [1,2]. Cataracts progress due to damage to the proteins in the lens of the eye, with the lens, becoming translucent or opaque. The main types of cataracts depend on their location in the lens, including nuclear, cortical, and posterior subcapsular [3,4]. There are non-modifiable, difficult, and uncontrolled factors that may increase the risk of developing cataracts. These include age, family history, and ethnicity [5-7]. Some studies also suggest that women may be at a slightly higher risk than men.

Numerous studies indicate that there is a link between dietary habits and the development of cataracts. The antioxidant properties of vitamins C and E could have a protective role against the development and progression of cataracts [8-10]. Also, it is suggested that the carotenoids lutein and zeaxanthin, which are antioxidants, may have a protective role against cataracts [11,12].

Recently, significant evidence has associated abnormal glucose metabolism with an increased likelihood of cataracts [13-16]. Many studies have focused on the probable association between carbohydrate intake and cataracts; nevertheless, the results are questionable [17-19]. The aim of the current study was to assess the evidence of an association between patients’ dietary habits, in terms of quantity and quality, and the risk of cataracts in the Aseer Region, Saudi Arabia.

## Materials and methods

A retrospective study was conducted targeting all accessible patients diagnosed with cataracts at Aseer Central Hospital, which is the main tertiary hospital in Abha, the capital of the southern region of Saudi Arabia, during the period from August 8, 2019, to March 3, 2021, and at Khamis Mushayt General Hospital during the period from June 11, 2018, to March 3, 2021.

Adults with cataracts who visited hospitals were tracked down by searching the medical record system with the term "cataract." Two authors extracted data from the medical record and patients. After the data have been extracted, the patient will be contacted via mobile phone to invite them to participate in the study.

If the patient agrees to participate, an email link with an encrypted and high-security electronic signature will be provided to him or her to get his or her consent.

After the consent is electronically signed, the patient will be asked about socio-demographic data, including age, gender, education, work type, and marital status. Also, the type of cataract diagnosed for the study patients was recorded along with associated risk factors for cataracts, including chronic diseases, trauma, eye surgery, family history, and all necessary clinical information, which will be recorded in a secured electronic data capture system (Castor EDC®). Patients aged less than 18 years and those who refused to participate in the study were excluded.

Data analysis

After data were extracted, they were revised, coded, and fed to statistical software IBM SPSS version 22 (SPSS, Inc., Chicago, IL, USA). Two-tailed tests were used for the statistical analysis. A P-value of less than 0.05 was statistically significant. A descriptive analysis using frequency distribution with percent was conducted for patients’ personal data, type of cataract, risk factors for cataracts, and dietary habits. Cross-tabulation was performed to assess the relationship between the type of cataract diagnosed among patients and their dietary habits. The significance of relationships was tested using a chi-square test and an exact probability test for small frequency distributions.

## Results

A total of 140 patients with cataracts who fulfilled the inclusion criteria were included in the study. Patients’ ages ranged from 21 to 65 years, with a mean age of 54.2 ± 12.9 years old. Seventy-six (54.3%) patients were female, and 134 (95.7%) were Saudis. As for work, 103 (73.6%) patients were not working or retired; 33 (23.6%) were non-healthcare workers; and 4 (2.9%) were healthcare workers. Considering educational level, 100 (71.4%) had below a secondary level of education, 20 (14.3%) had a secondary educational level, and 20 (14.3%) had a university education. A total of 128 (91.4%) were married. As for patients’ BMI, 14 (10%) had normal weight, 13 (9.3%) were overweight, and 12.2% were obese, while 96 (68.6%) did not know their BMI (Table 1).

**Table 1:** Socio-demographic data of patients with cataracts, Aseer Region, Saudi Arabia

Socio-demographic data	No	%
Age in years		
21–30	5	3.6%
31–40	12	8.6%
41–50	13	9.3%
>50	110	78.6%
Gender		
Male	64	45.7%
Female	76	54.3%
Nationality		
Saudi	134	95.7%
Non-Saudi	6	4.3%
Work		
Not working	103	73.6%
Non-health care worker	33	23.6%
Health care worker	4	2.9%
Educational level		
Below secondary	100	71.4%
Secondary	20	14.3%
University/above	20	14.3%
Marital status		
Unmarried	12	8.6%
Married	128	91.4%
BMI		
Normal	14	10.0%
Overweight	13	9.3%
Obese	4	2.9%
Morbid obesity	13	9.3%
Do not know	96	68.6%

**Figure 1:**
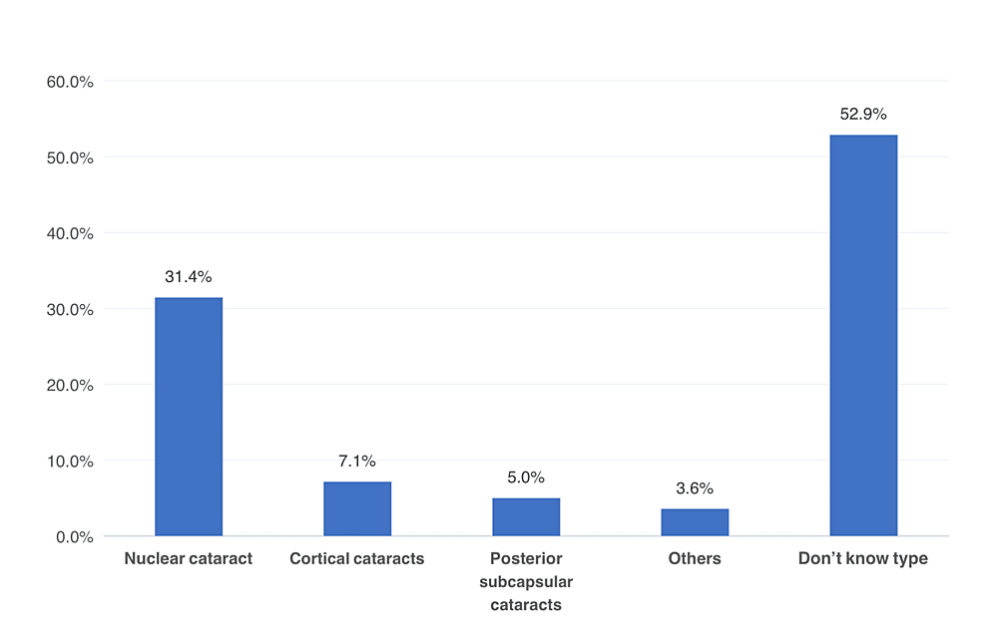
Type of cataracts among study patients, Aseer Region, Saudi Arabia

The type of cataract among study patients in Saudi Arabia was shown in Figure 1. The most identified type of cataract was nuclear cataract (31.4%), followed by cortical cataract (7.1%) and posterior subcapsular cataract (5%), and 2.6% had other types, including traumatic cataract, while 52.9% of the patients did not know their type of cataract.

Risk factors for cataracts among study patients in the Aseer Region, Saudi Arabia were given in Table 2. It was found that 12.1% of the study patients were smokers, 44.3% were diagnosed with hypertension, and 45% were diabetic, which was type 1 diabetes mellitus (T1DM) among 36.5% and type 2 diabetes mellitus (T2DM) among 52.4%. A family history of congenital cataracts was reported among 12.9%, and 12.9% had a history of eye trauma. Medication that contains cortisone was taken by 15.7% of the study patients, and 40.7% had a history of eye surgery. Vitamin D deficiency was reported among 7.9% of the study patients, and 34.3% had eye allergy.

**Table 2:** Risk factors for cataracts among study patients, Aseer Region, Saudi Arabia

Risk factors	No	%
Are you a smoker?		
Yes	17	12.1%
No	123	87.9%
Do you suffer from hypertension?		
Yes	62	44.3%
No	78	55.7%
Do you suffer from diabetes?		
Yes	63	45.0%
No	77	55.0%
Type of DM (n=63)		
T1DM	23	36.5%
T2DM	33	52.4%
Pre-diabetes	7	11.1%
Do you have anyone in your family with congenital cataracts?		
Yes	18	12.9%
No	122	87.1%
Do you suffer from any trauma or eye disease?		
Yes	18	12.9%
No	122	87.1%
Do you use any type of medication that contains cortisone?		
Yes	22	15.7%
No	118	84.3%
Have you ever had eye surgery?		
Yes	57	40.7%
No	83	59.3%
Do you suffer from vitamin C deficiency?		
Yes	11	7.9%
No	27	19.3%
Don’t know	102	72.9%
Do you have an allergy to your eyes from anything?		
Yes	48	34.3%
No	92	65.7%

**Figure 2:**
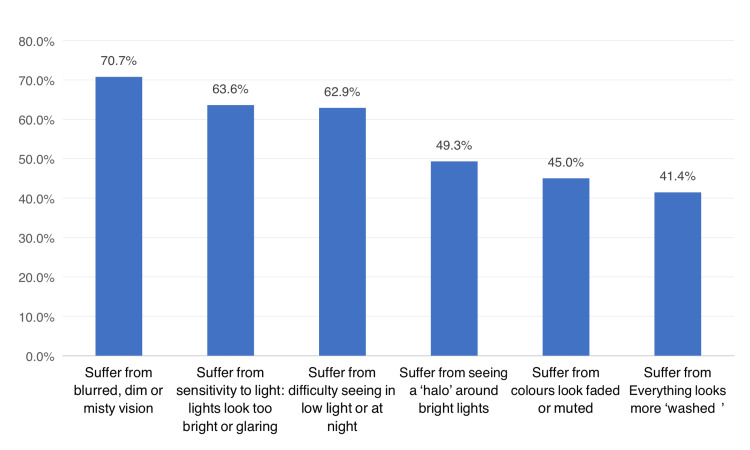
Clinical symptoms among study patients with cataracts, Aseer Region, Saudi Arabia

Figure 2 shows clinical symptoms among study patients with cataracts in the Aseer Region, Saudi Arabia. The most reported symptoms were blurred, dim, or misty vision (70.7%), followed by sensitivity to light (lights look too bright or glaring; 63.6%), difficulty seeing in low light or at night (62.9%), seeing a "halo" around bright lights (49.3%), colors looking faded or muted (45%), and everything looking more "washed" (41.4%).

Table 3 shows the dietary habits of study patients with cataracts in the Aseer Region, Saudi Arabia. It was found that 37.1% of the study patients have vegetables one time per week, while 25.7% have vegetables three times per week. Eating fruits or having juice was reported as once per week among 40% of the study patients and three times per week among 20%. Eating fish that contained amino acids was reported once per week among 37.9% of the study patients, while 51.4% did not have fish at all. Dairy product intake of three times per week was reported among 50.7% of the study patients, while 15% had dairy products only once per week. A diet containing legumes and nuts was reported among 58.6% of the study patients, and 31.4% took nutritional supplements or vitamins. Eating a lot of sugary snacks was reported by 34.3% of the study patients, and 11.4% had a lot of soft drinks.

**Table 3:** Dietary habits of study patients with cataracts, Aseer Region, Saudi Arabia

Dietary habits	No	%
How many times do you eat vegetables in a week?		
3 times/week	36	25.7%
2 times/week	40	28.6%
Once/week	52	37.1%
Don’t have	12	8.6%
How many times do you eat fruits or drink a glass of juice per week?		
3 times/week	28	20.0%
2 times/week	39	27.9%
Once/week	56	40.0%
Don’t have	17	12.1%
How often do you eat fish that contain amino acids?		
3 times/week	5	3.6%
2 times/week	10	7.1%
Once/week	53	37.9%
Don’t have	72	51.4%
Frequency that your diet contains milk and dairy products/week?		
3 times/week	71	50.7%
2 times/week	34	24.3%
Once/week	21	15.0%
Don’t have	14	10.0%
Does your diet contain legumes and nuts?		
Yes	82	58.6%
No	58	41.4%
Do you take any nutritional supplements or vitamins?		
Yes	44	31.4%
No	96	68.6%
Do you eat a lot of sugary snacks?		
Yes	48	34.3%
No	92	65.7%
Do you drink a lot of soft drinks?		
Yes	16	11.4%
No	124	88.6%

Table 4 shows the relationship between patients’ dietary habits and the type of cataract. Nuclear cataract was diagnosed among 32.8% of patients with frequent vegetable intake compared to 30.1% of others with infrequent intake, and posterior subcapsular type was diagnosed among 10.4% of those with frequent vegetable intake versus none with infrequent intake (P=0.042). Additionally, 32.8% of patients with nuclear cataracts reported infrequent fish intake compared to 20% of those with frequent fish meals per week (P=0.032). Other dietary habits showed a non-significant relationship with the type of cataract.

**Table 4:** The relationship between patients’ dietary habits and type of cataracts P: Pearson X^2^ test; ^$^exact probability test; ^#^patients with unsure type were excluded; *P < 0.05 (significant).

Dietary habits	Cataract^#^								p-value
	Nuclear cataract		Cortical cataracts		Posterior subcapsular cataracts		Others		
	No	%	No	%	No	%	No	%	
Vegetable’s intake									0.154^$^
Infrequent/none	22	34.4%	4	6.3%	0	0.0%	2	3.1%	
Frequent	22	28.9%	6	7.9%	7	9.2%	3	3.9%	
Fruits and juice intake									0.042*^$^
Infrequent/none	22	30.1%	5	6.8%	0	0.0%	4	5.5%	
Frequent	22	32.8%	5	7.5%	7	10.4%	1	1.5%	
Fish containing amino acids intake									0.032*^$^
Infrequent/none	41	32.8%	8	6.4%	6	4.8%	5	4.0%	
Frequent	3	20.0%	2	13.3%	1	6.7%	0	0.0%	
Frequency that your diet contains milk and dairy products/week?									0.965^$^
3 times/week	22	31.0%	6	8.5%	3	4.2%	3	4.2%	
2 times/week	12	35.3%	1	2.9%	3	8.8%	1	2.9%	
Once/week	6	28.6%	2	9.5%	1	4.8%	0	0.0%	
Don’t have	4	28.6%	1	7.1%	0	0.0%	1	7.1%	
Do you eat a lot of sugary snacks?									0.991
Yes	15	31.3%	3	6.3%	2	4.2%	2	4.2%	
No	29	31.5%	7	7.6%	5	5.4%	3	3.3%	
Do you drink a lot of soft drinks?									0.164^$^
Yes	3	18.8%	0	0.0%	0	0.0%	0	0.0%	
No	41	33.1%	10	8.1%	7	5.6%	5	4.0%	
Do you take any nutritional supplements or vitamins?									0.370
Yes	12	27.3%	2	4.5%	1	2.3%	3	6.8%	
No	32	33.3%	8	8.3%	6	6.3%	2	2.1%	

## Discussion

The eye is the organ through which people perceive their surroundings by changing light into electrical signals. Focusing light on the retina is the job of the eye lens, confirming that both close and distant objects can be clearly seen [20]. To perform its function adequately, the lens must be transparent. The high density of lenses’ structural proteins, combined with their spatial arrangement in a repeating matrix, results in a body with a nearly uniform refractive index, making it transparent [21]. In the eye, oxidation affects proteins and fats in the lens to the degree that the lens may be damaged and become cloudy, causing cataracts. Preventing damage by eating healthy foods, mainly those containing antioxidants, may help slow down this process [22].

The current study aimed to assess the relationship between nutrient intake and developing cataracts among patients in the Aseer Region, Saudi Arabia. The study revealed that most patients with cataracts were aged over 50 years, and more than half of them were female. Also, the vast majority of patients had a low level of education (below secondary) and were not working (mostly retired or aged). Regarding the type of cataract, the most diagnosed among one-third of the patients was nuclear cataract, followed by other, less frequent types, including cortical cataracts and posterior subcapsular cataracts. These types are the most common among patients worldwide [23,24].

Regarding risk factors reported among the study patients, the most reported were old age (>50 years); diabetes mellitus, especially T1DM; hypertension; a history of eye surgery; and eye allergy. Other less frequent risk factors were reported, including a family history of congenital cataracts, taking drugs containing corticosteroids, and vitamin C deficiency. The majority of patients did not know whether they had vitamin C deficiency, which explains the low percentage of those who said they had. The reported risk factors are the main factors reported globally for cataracts through different reviewed research studies [25-28].

Considering the dietary habits of study patients with cataracts, the study showed that the vast majority of patients (91.4%) had vegetables at least once per week, 90% of the patients had a diet containing milk and dairy products, and 87.9% had fruits and juice at least once per week. Also, more than two-thirds of the patients took nutritional supplements or vitamins, and about 40% had a diet containing legumes and nuts. On the other hand, more than half of the patients did not have fish containing amino acids at all, which have a significant role in developing eye diseases, including cataracts [29,30]. Also, a high frequency of soft drink intake was reported among more than three-quarters of the study patients. In addition, two-thirds eat a lot of sugary snacks.

Lee et al. [31] reported that cataracts were associated with some type of fruit and vegetable intake. The controls had more vegetables and fruits than the cases, and the lower total intake of vegetables and fruits increased the risk of cataracts by up to 1.7-fold (95% confidence interval: 1.06-2.71). Sella and Afshari [32] recommend that a diet rich in fruits and vegetables and a high dietary intake of vitamins A, C, D, E, and K1 may minimize the risk of age-related cataracts. During the last century, two studies determined that milk consumption may reduce the risk of cataract formation [33,34]. Nevertheless, in a more recent case-controlled study [35], dairy products did not seem to change the risk of cataracts.

## Conclusions

There is currently an inquiry to endorse or exclude a specific diet or dietary intake that may reduce or even prevent the development and progression of cataracts. As to the positive association between nutrition and cataracts, it appears beneficial for people to have some antioxidants in their daily food. Eating more fruits and vegetables with more vitamin intake is recommended by the National Cancer Institute. The results should be considered carefully, and more studies are recommended to prove the proposed evidence. Also, prospective studies are recommended to identify the types of cataracts most affected by specific types of food, dietary patterns, or environmental influences, which will improve patients’ quality of life and help reduce the extensive load of cataracts on public health resources.
